# Different lactate metabolism subtypes reveal heterogeneity in clinical outcomes and immunotherapy in lung adenocarcinoma patients

**DOI:** 10.1016/j.heliyon.2024.e30781

**Published:** 2024-05-07

**Authors:** Jing Zhang, Yun Bao, Yang Li, Xin Shi, Xiangyu Su, Xuejun He

**Affiliations:** a Department of Oncology, The Second People's Hospital of Taizhou Affiliated to Medical College of Yangzhou University, Taizhou, 225500, PR China; b Department of Oncology, Zhongda Hospital, School of Medicine, Southeast University, Nanjing, 210009, Jiangsu, PR China

**Keywords:** Lung adenocarcinoma (LUAD), Lactate metabolism, Immune microenvironment, Drug resistance, SLC16A3

## Abstract

**Background:**

The excessive accumulation of lactate within the tumor microenvironment (TME) has been demonstrated to facilitate tumor advancement and evade the immune system. Nonetheless, the metabolic status of lactate in lung adenocarcinoma (LUAD) remains uncertain.

**Method:**

By analyzing the transcriptome profile of patients with LUAD, we created a lactate metabolism score (LMS) to predict survival. We then conducted a comprehensive examination of the biological functions and immune infiltration among different LMS patient groups. Moreover, we assessed the LMS predictive efficacy in chemotherapy and immunotherapy. Finally, we validated the detrimental phenotypic effects of SLC16A3 on LUAD cell lines (PC9 and A549) through in vitro experiments. We collected clinical samples to assess the prognostic impact of SLC16A3.

**Results:**

We constructed an LMS model with 6 lactate metabolism regulatory factors using LASSO regression. The high LMS model indicates worse clinical outcomes for LUAD patients. High LMS patients are associated with metabolic dysregulation and increased infiltration of M0 and M1 macrophages. Low LMS patients are related to upregulated citric acid metabolism pathways and memory immune cells. High LMS patients are suitable for traditional chemotherapy, while patients with low LMS are more likely to benefit from immunotherapy. Lastly, downregulating SLC16A3 significantly reduces the proliferative and invasive capabilities of LUAD cell lines. Clinical cohort shows that patients with high expression of SLC16A3 have a worse prognosis.

**Conclusions:**

The LMS model constructed based on the lactate metabolism pathway displays high effectiveness in predicting the outcome of patients with LUAD. LMS can offer direction regarding chemotherapy as well as immunotherapy in LUAD.

## 1 Introduction

In the present global context, out of all the cancer types, lung cancer possesses the highest incidence as well as mortality rate, thus emerging as the main factor contributing to fatalities from cancer [1]. Lung adenocarcinoma (LUAD) constitutes the most prevalent lung cancer's histological classification, which accounts for a substantial proportion of cases. Numerous epidemiologists and clinical research cohorts primarily attribute the emergence as well as development of LUAD to various environmental variables in addition to genetic changes [[2], [3], [4]]. The previous theory that solely emphasized environmental factors has been contradicted by the presence of a significant number of non-smokers with LUAD, instead highlighting profound genetic alterations. Currently, genetic-based treatment approaches mainly revolve around targeted therapy and immunotherapy [5]. However, it is unfortunate that most patients undergoing targeted therapy exhibit tendencies of drug resistance, even though immunotherapy only helps a small percentage of patients. Hence, exploring and developing robust tools for predicting prognosis and treatment response is crucial, as it will further promote precise diagnosis and personalized treatment.

Lactic acid is an endogenous organic acid that is normally present in the human body at low concentrations but can significantly increase in certain diseases, including cancer [6]. Its role in cancer is complex, as it affects various aspects of tumor biology. In normal circumstances, cells generate energy through oxidative phosphorylation, an oxygen-dependent process. However, anaerobic glycolysis is a common method used by cancer cells to generate energy, converting glucose into lactic acid and releasing a small amount of energy [7]. Remarkably, glycolysis remains the primary energy source for cancer cells, even in situations where there is sufficient oxygen available [8]. The aberrant accumulation of lactic acid in the tumor microenvironment (TME) leads to substantial changes in various phenotypic features [9]. For instance, lactic acid can disrupt the acid-base balance of the TME, regulate neighboring cells and tissues, in addition to encourage the survival and proliferation of cancer cells [10]. Additionally, lactic acid secretion by tumor cells can induce the release of pro-angiogenic factors, thereby stimulating angiogenesis, supplying more nutrients and oxygen to the tumor, and facilitating its growth and spread [11]. Furthermore, elevated lactic acid levels impair the activity of crucial immune cells, for instance, T cells as well as natural killer cells, compromising their ability to eliminate tumor cells and avoiding immune monitoring by cancer cells [12]. Moreover, high levels of lactic acid influence drug metabolism and elimination within tumor cells, resulting in resistance to chemotherapy and radiation therapy [13]. Therefore, lactic acid's effects on different types and stages of cancer may vary. Elucidating the role of lactic acid in cancer may aid in the creation of more potent treatment strategies and interventions, ultimately improving the cancer patients' quality of life as well as survival rate.

The objective of this particular research is to investigate the correlation between lactic acid levels and LUAD by conducting thorough bioinformatics analysis and in vitro experiments. We employed four sets of transcriptomic data acquired from the TCGA and GEO databases. To quantitatively assess the levels of lactic acid in patients, we developed a lactic metabolism score (LMS) using the Cox regression and LASSO regression methods. By analyzing immune infiltration as well as functional enrichment, we sought to elucidate the potential underlying mechanisms through which LRS influences the progression of LUAD. Moreover, we assessed the predictive capability of LRS for chemotherapy and immunotherapy. Lastly, we carried out in vitro experiments to validate the promotive impact of SLC16A3 gene knockout on the proliferation and invasive capacity of LUAD cell lines.

## 2 Methods

### 2.1 Acquisition and pre-processing of data

We acquired FPKM RNA sequencing data for the transcriptome, Mutect2 mutation data, GISTIC2.0 copy number variation (CNV) data, as well as relevant clinical information for patients with TCGA-LUAD derived from the TCGA database (https://portal.gdc.cancer.gov/) utilizing the GDC API. Patients who lacked clinical information or experienced loss of follow-up were omitted from the sample set, which results in the collection of 492 LUAD samples in total. The training dataset was the original FPKM sequencing data that had been TPM-normalized. From the GEO repository, we retrieved three established LUAD datasets, namely GSE30219, GSE42127, and GSE72094. To mitigate batch effects arising from the microarray chips, these three GEO datasets were combined using the “combat” function in the “sva” package and performed log2 normalization on the data [14]. Ultimately, we curated 615 LUAD metadata with comprehensive clinical information to serve as the validation dataset. We specifically retrieved a cohort related to lung cancer immune therapy, resulting in the inclusion of GSE135222, which comprised 27 cases that received anti-PD-1 therapy [15]. Furthermore, to substantiate the predictive effect of LMS on immune therapy, two large-scale and well-established immune therapy cohorts, namely Imvigor210 and nature-SKCM, were incorporated into this study [16,17]. We collected 24 lactate metabolism genes (LMGs) from MSigDB database (https://www.gsea-msigdb.org/gsea/msigdb/), the detailed list of lactate metabolism genes is provided in Table S1.

### 2.2 Development of a scoring model for lactate metabolism

We employ two methods to construct a robust LMS model. First, we utilize uni-variable Cox regression analysis to determine factors within the gene set corresponding to lactate metabolism that demonstrate significant prognostic efficacy. Next, we input these significant prognostic lactate factors into a LASSO penalized Cox regression pipeline, using an appropriate penalty factor to identify key prognostic lactate factors and generate gene coefficients. The final LMS model is generated using the following formula 1:

(1) $$LMS=\sum iCoefficient\left({mRNA}_{i}\right)\times Expression\left({mRNA}_{i}\right)$$

### 2.3 Assessing the clinical application potential of the lactate metabolism score model

We utilized the “survminer” package to plot survival curves related to LMS, distinguishing high LMS patients from low LMS patients as per the median LMS value. Subsequently, we generated LMS-related ROC curves using the “pROC” package and “survivalROC” package. Furthermore, we have performed both univariate as well as multivariate regression analyses in order to examine the prognostic capacity of LMS for the overall survival (OS) rate among individuals diagnosed with LUAD. Finally, with the assistance of the “rms” package, we developed an LMS-related nomogram for individualized risk stratification in LUAD patients.

### 2.4 Analysis the biological functions and immune infiltration related to LMS

We performed functional annotation and enrichment analysis of differentially expressed genes (DEGs) between the high and low LMS groups using the online tool platform Metascape (www.metascape.org/) [18]. A fold change threshold of >2 and a *P*-value <0.05 were used to filter DEGs. Subsequently, we proceeded to perform a GSEA analysis between the high and the low LMS groups using the “clusterProfiler” package to identify significantly active KEGG pathways in different LMS groups [19]. We evaluated tumor purity and corresponding immune scores with respect to individual LUAD samples using the “estimate” algorithm [20]. By utilizing previously published immune cell gene lists and their corresponding expression profiles, we assessed the relative infiltration abundance of 24 immune cells using the “cibersort” algorithm [21]. Lastly, we assessed the correlation between LMS and tumor-related pathways using the ssGSEA algorithm as per the “HALLMARK” gene set.

### 2.5 Analysis of the differences in treatment response among different LMS

The half-maximal inhibitory concentration (IC50) value regarding the LUAD chemotherapy agents was estimated for different LMS patients to evaluate LMS's predictive ability for traditional chemotherapy [22]. Ten-fold cross-validation was performed to ensure prediction result accuracy. DEGs between various LMS groups were identified as potential targets for novel targeted therapies. The top 150 DEGs were submitted to the Cmap database (https://clue.io/) to discover targeted drugs associated with LMS. To evaluate the response rates of different LMS patients to immunotherapy, the Immunophenoscore (IPS) was first estimated based on the expression pertaining to the immune cell-related markers in the samples [23]. A higher IPS value indicates a greater possibility of an immunotherapy response in patients [23]. Subsequently, the “TIDE” algorithm was employed to project the immune checkpoint inhibitor response rates of various LMS patients, considering the levels of T cell exhaustion in the sequencing samples [24]. Finally, LMS was generated using the same formula in the previously mentioned three immunotherapy datasets to evaluate its predictive accuracy in real-world immunotherapy cohorts.

### 2.6 Cell culture and siRNA transfection

The human lung cancer cell lines PC9 and A549 were acquired from Procell (Procell, Wuhan, China). Both cell lines were cultivated in DMEM medium (Bological, Israel) and maintained in a CO2-filled constant temperature incubator at 37 °C. Heat-inactivated 10 % fetal bovine serum (FBS) as well as 1 % penicillin were supplemented to the DMEM medium. The Lipo2000 kit (Invitrogen, USA) was employed as per the manufacturer's protocol for transiently transfecting siRNA, specifically targeting SLC16A3 to transiently reduce the target gene's expression. Two siRNA sequences targeting SLC16A3 were utilized: si-SLC16A3-1 CGCACGTCTGCTGATGGCCTGGCTG, si-SLC16A3-2 CCGTGGCTCTGTCCTCGCCAGCTTT. Following the transfection, the cells were cultured for 48 h, followed by qRT-PCR and Western blot experiments to examine the knockdown effect of SLC16A3 at the transcriptional and protein levels.

### 2.7 Detection of cell proliferation level

A colony formation assay was conducted to assess the influence of SLC16A3 knockdown on the proliferative capacity of LUAD cell lines. After 48 h of cultivation, single-cell suspensions were obtained, and a 6-well plate had about 1000 cells seeded in each well. The cells were cultured in standard DMEM medium, with medium changes performed every three days. After the LUAD cells showed signs of colony formation, they were fixed with 4 % paraformaldehyde and stained with crystal violet solution, and subsequently subjected to cell counting via microscopy imaging.

### 2.8 Cell invasion and migration ability detection

Transwell experiments were conducted using two types of LUAD cells: SLC16A3 knockdown cells and control cells. Their invasion and migration capabilities were assessed. Matrigel was introduced into the chambers of the Transwell assay kit to measure invasion, while migration was evaluated in the chambers without Matrigel. The number of invading cells was quantified via ImageJ software. Once the fusion rate of PC9 and A549 cells reached 90 % in the DMEM culture dish, the cells were harvested and starved in serum-free DMEM medium for 24 h. Using a 200 μl pipette tip, a scratch was deliberately made on the starved cells, which were then washed with warm serum-free medium to eliminate cellular debris. After 48 h, the extent of wound healing at the scratch was assessed under a microscope, and the wound healing ratio was computed using ImageJ software to evaluate the migratory capacity of the cells.

### 2.9 Immunohistochemistry and staining intensity assessment

Surgical tissue sections from 30 individuals with lung adenocarcinoma from Sichuan Provincial People's Hospital were collected for this study, and every participant provided informed consent by signing a consent form. Sample collection was accepted by the Sichuan Provincial People's Hospital Ethics Committee. In order to repair antigens in tissue sections, citrate buffer (pH 6.0) was applied initially at room temperature, followed by sealing with goat serum for 30 min. A SLC16A3 primary antibody (1:100, ab308529, abcam) was applied to the sections and incubated them for an entire night at 4 °C. Subsequently heated and incubated with secondary antibody (ab205718, abcam) at room temperature for 30 min.

Staining was carried out utilizing 3, 3- diaminobenzidine tetrahydrochloride (DAB) (ZSGB-BIO, Beijing). Imaging was visualized using a microscope to assess immunoreactivity proximity based on histochemical scoring (H-score) under a 10× objective, and a 40× objective to obtain more detailed staining information.

### 2.10 Statistical analysis

The analysis in this manuscript utilized the R environment (Version 4.1.1). Student's t-test evaluated the variations in continuous variables between two groups, Fisher's exact test examined significant differences between categorical variables, and Spearman's correlation coefficient evaluated the correlation among continuous variables. P < 0.05 was designated as the statistical significance threshold.

## 3 Results

### 3.1 The landscape of lactate metabolism genes in LUAD

**Fig. 1:**
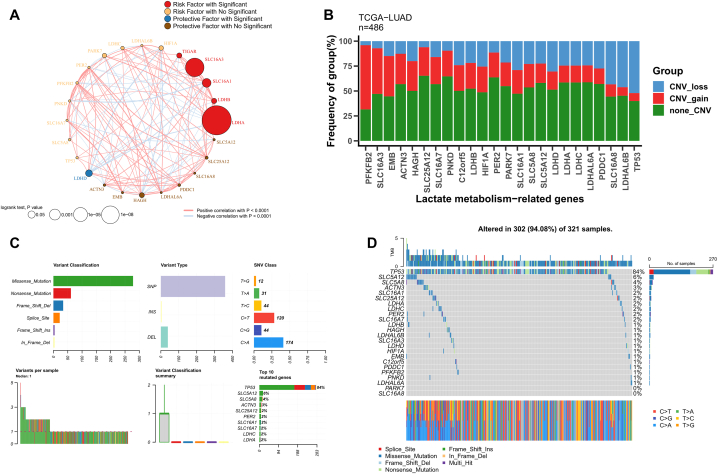
The landscape of lactate metabolism genes in LUAD. (A) The correlation network and prognostic efficacy of 24 lactate metabolism genes. (B) The summary of copy number variations in 24 lactate metabolism genes. (C) The summary of single nucleotide variants in 24 lactate metabolism genes. (D) Oncoplot demonstrates mutational profiles of 24 lactate metabolism genes.

We identified the prognostic impact of 24 lactate metabolism genes (LMGs) using univariate Cox regression analysis and constructed their correlation network. The results, as shown in Fig. 1A, revealed that 5 LMGs (LDHA, LDHB, SLC16A1, SLC16A3, TIGAR) were significantly associated with risk, while one LMG (LDHD) was identified as a significant protective factor. Apart from LDHD, the other 23 LMGs demonstrated significant positive correlations. Subsequently, we analyzed changes to LMG copy numbers and discovered that PFKFB2 exhibited the highest frequency of amplification, while TP53 and LDHAL6B were the most frequently deleted genes (Fig. 1B). We also investigated LMG-related single nucleotide variations. The results indicated that among LMGs, TP53 had the highest mutation rate (Fig. 1C) and 1D displayed a detailed mutation profile of LMGs.

### 3.2 Construction and verification of LMS

**Fig. 2:**
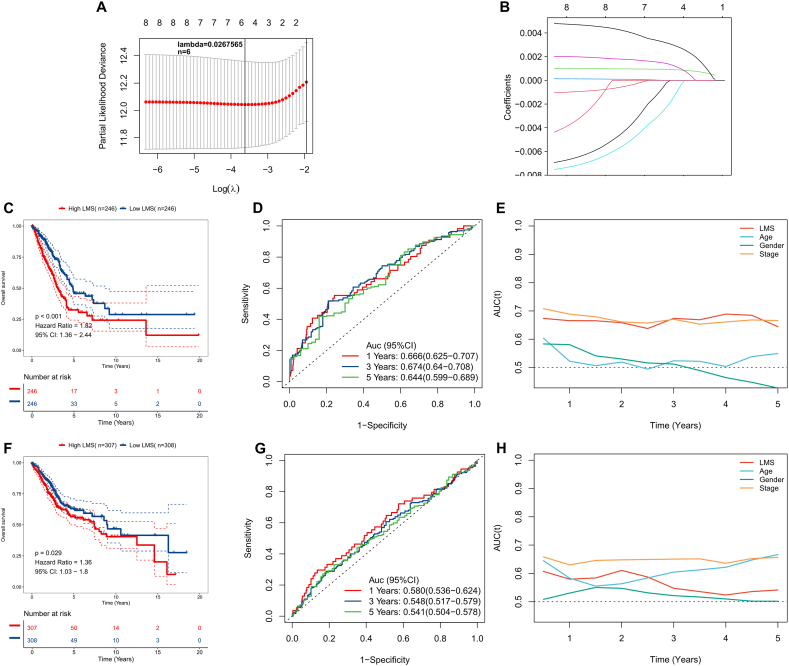
Construction and verification of LMS. (A) Select the best penalty factor λ to construct the final LMS model. (B) Convergence of 6 genes according to the optimal λ to construct the LMS model. (C) KM survival curves for the high-LMS and low-LMS patients in the TCGA-LUAD cohort. (D) ROC curve of LMS for predicting OS in the TCGA-LUAD cohort. (E) Time ROC curve of LMS for predicting OS in the TCGA-LUAD cohort. (F) KM survival curves for the high-LMS and low-LMS patients in the GEO-LUAD cohort. (G) ROC curve of LMS for predicting OS in the GEO -LUAD cohort. (H) Time ROC curve of LMS for predicting OS in the GEO-LUAD cohort.

To avoid omission, we selected the predicted LMGs with P < 0.1 in univariate regression as candidate variables for the final LMS model. After five-fold cross-validation, the optimal penalty factor λ was determined to be 0.0267565 (Fig. 2A). The final LMS model converged to six genes (Fig. 2B). LMS was generated based on the formula: 0.003323*Exp(SLC16A3)+ 0.001517*Exp(SLC16A1)+ 0.00093*Exp(LDHA)+ 0.00002*Exp(LDHB)- 0.00098*Exp(HAGH)- 0.00291*Exp(LDHD), and the KM survival curve demonstrated that LUAD patients having high LMS values had significantly lower OS rates in both TCGA and GEO cohorts compared to patients having low LMS values (Fig. 2C and F). The ROC curve demonstrated satisfactory predictive performance of LMS in the TCGA cohort (AUC>0.65, refer to Fig. 2D). However, in the external validation cohort GEO, the performance of LMS was not superior (AUC<0.6, Fig. 2G). Moreover, in the TCGA cohort, LMS showed superior predictive efficacy compared to other clinical indicators (Fig. 2E), but in the GEO cohort, the advantage of LMS was not significant (Fig. 2H).

### 3.3 Constructing LMS-related risk stratification for LUAD patients

**Fig. 3:**
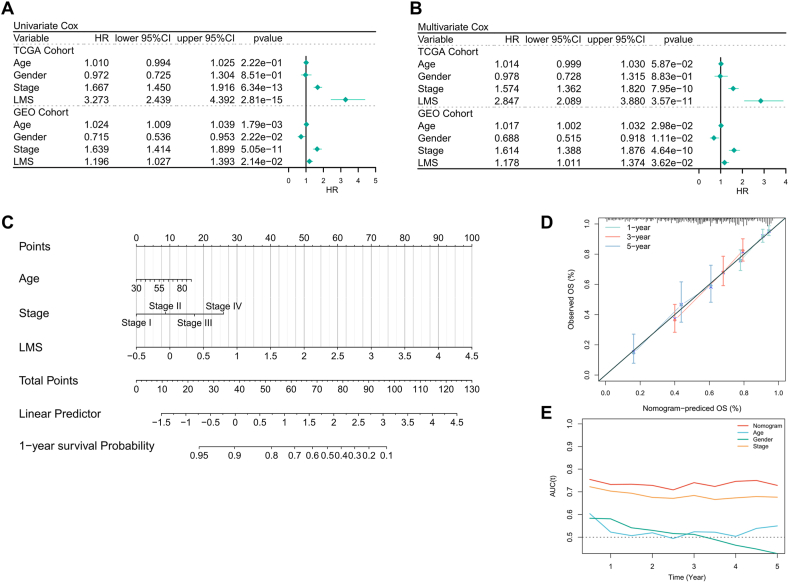
Constructing LMS-related risk stratification for LUAD patients. (A) Univariate Cox regression on LMS in TCGA-LUAD and GEO- LUAD cohorts; (B) Multivariate Cox regression analysis on LMS in TCGA-LUAD and GEO- LUAD cohorts. (C) Building a Nomogram based on LMS and clinical information for risk stratification of luad patients. (D) Calibration curves for the LMS-based nomogram. (E) The timeROC curves for the LMS-based nomogram and other clinical characteristics.

We initially evaluated the robustness and independence of LMS in predicting OS in LUAD patients. Univariate Cox regression analysis demonstrated that LMS could independently serve as a risk factor for patient OS across different LUAD cohorts (refer to Fig. 3A). Multivariate Cox regression analysis revealed that even after adjusting for age, gender, and tumor stage, LMS remained a reliable predictor of OS in LUAD patients (Fig. 3B). Subsequently, we combined patients' LMS and clinical information to construct a personalized risk stratification Nomogram based on lactate metabolism (Fig. 3C). By taking the clinical information and the LMS of individual patients into the Nomogram and calculating the total score, the patient's survival at 1, 3, as well as 5 years can be predicted (Fig. 3C). The Nomogram demonstrated excellent calibration for predicting 1-, 3-, as well as 5-year survival probabilities (refer to Fig. 3D). Ultimately, tROC analysis indicated that the Nomogram model exhibited superior predictive accuracy for the five-year timeframe compared to the use of clinical variables alone (Fig. 3E).

### 3.4 Dissecting LMS-related functional enrichment

**Fig. 4:**
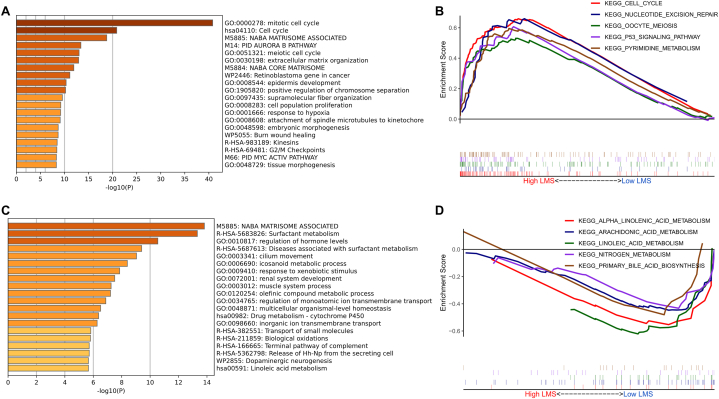
Dissecting LMS-related functional enrichment. (A) The bar plot showed the biological pathways of upregulated gene enrichment in the high LMS patients. (B) GSEA reveals Top5 pathways upregulated in patients with high LMS. (C) The bar plot showed the biological pathways of upregulated gene enrichment in the low LMS patients. (D) GSEA reveals Top5 pathways upregulated in patients with low LMS.

We aimed to investigate the biological heterogeneity among various LMS patients, so we first conducted functional enrichment analysis on highly expressed genes in different LMS groups. The results demonstrated that the majority of the upregulated genes in high LMS patients were involved in regulating the cell cycle, extracellular matrix, and hypoxia (Fig. 4A). As per the GSEA results, the P53 pathway, purine metabolism, mitosis, the cell cycle, as well as mitosis were the main pathways that were upregulated in high LMS tumor samples (Fig. 4B). In low LMS patients, the upregulated genes mainly functioned in regulating drug metabolism pathways (Fig. 4C). Finally, we found that the upregulated pathways in the low LMS group were essentially associated with metabolism, including alpha linoleic acid, linoleic acid, and nitrogen (Fig. 4D).

### 3.5 Dissecting LMS-related immune infiltrates

**Fig. 5:**
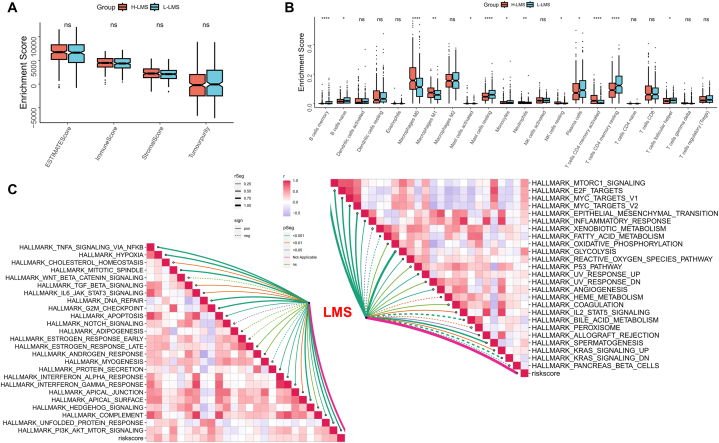
Dissecting LMS-related immune infiltrates. (A) Box plot showing the difference in ESTIMATE scores between LUAD patients with different LMS levels. (B) Box plot showing the difference in immune cell infiltrating between LUAD patients with different LMS levels. (C) Correlation network of LMS and 50 cancer hallmark pathways in LUAD patients.

First, we assessed the overall composition of the TME in various LMS groups using ESTIMATE. The findings revealed no substantial variations in immune scores and tumor purity among the different LMS subtypes (Fig. 5A). Subsequently, we conducted a detailed evaluation of the infiltration abundance differences of 24 different immune cells in different LMS groups. The findings showed that the low LMS group possessed higher levels of infiltration with B cells, plasma cells, as well as CD4T cells, whereas the high LMS group possessed higher levels of infiltration with M0 and M1 macrophages (refer to Fig. 5B). Lastly, we assessed the correlation between 50 cancer markers and LMS. The findings showed that there was a substantial positive correlation between LMS and classical cancer signaling pathways such as E2F, MYC, MTOR, P53, and KRAS (Fig. 5C).

### 3.6 LMS can predict the response to conventional chemotherapy

**Fig. 6:**
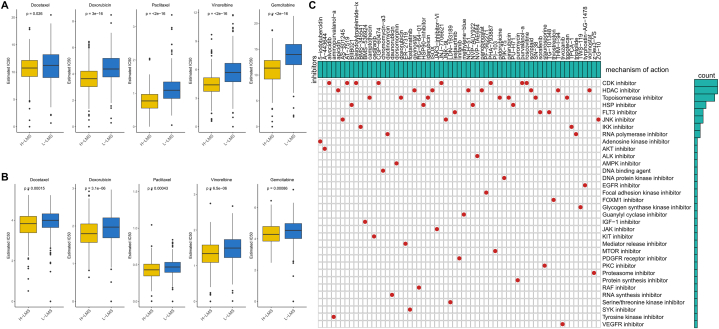
Predictive efficacy of LMS for conventional chemotherapy. (A) Box plot showing the difference in sensitivity of LUAD patients with different LMS levels to the five chemotherapeutic agents in the TCGA-LUAD cohort. (B) Box plot showing the difference in sensitivity of LUAD patients with different LMS levels to the five chemotherapeutic agents in the GEO-LUAD cohort. (C) Oncoplot shows 63 novel targeted drugs that may target lactate metabolism and their targets of action.

First, we evaluated the differences in response rates to conventional lung cancer chemotherapy drugs among different LMS patients. The findings indicated that patients with high LMS were more sensitive to five chemotherapy strategies (Fig. 6A). In an external validation cohort, it was also found that high LMS patients demonstrated increased sensitivity to all five lung cancer chemotherapy strategies (Fig. 6B). Subsequently, we identified novel targeted drugs related to LMS. A total of 63 potential novel targeted drugs were identified (Fig. 6C). Among them, the most promising lactate-related drug targets were the CDK and HDAC signaling pathways.

### 3.7 LMS can identify LUAD patients who benefit from immunotherapy

**Fig. 7:**
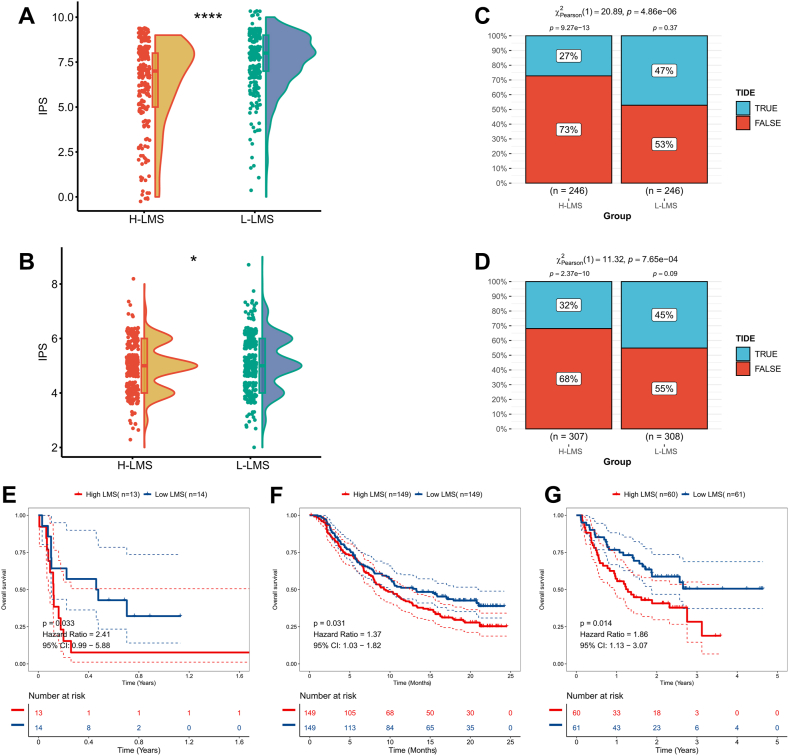
Predictive efficacy of LMS for immunotherapy. (A) The violin plot shows the differences in IPS in TCGA-LUAD patients with different LMS levels. (B) The violin plot shows the differences in IPS in GEO-LUAD patients with different LMS levels. (C) The bar chart statistics the response rate of patients with different LMS levels to immunotherapy in the TCGA-LUAD cohort. (D) The bar chart statistics the response rate of patients with different LMS levels to immunotherapy in the GEO-LUAD cohort. (E) KM survival curves for the high-LMS and low-LMS patients in the GSE135222 cohort. (F) KM survival curves for the high-LMS and low-LMS patients in the Imvigor210 cohort. (G) KM survival curves for the high-LMS and low-LMS patients in the Nature-SKCM cohort.

First, the overall response rate of various LMS patients to immunotherapy was assessed using the IPS index. According to the findings, patients with lower LMS scores possessed higher IPS scores, which suggested that they would benefit more from immunotherapy (Fig. 7A). In the external validation cohort, we also observed a significant increase in IPS scores for low LMS patients (Fig. 7B). Subsequently, we utilized the TIDE algorithm to assess the differential response rates of different LMS patients to immune checkpoint inhibitors. The results showed significantly increased response rates for low LMS patients in both LUAD cohorts (Fig. 7C and D). Finally, we analyzed LMS in three different immunotherapy cohorts and evaluated the predictive effect of LMS using survival curves. The results demonstrated that high LMS patients possessed significantly lower overall survival rates in these three immunotherapy cohorts (Fig. 7E–G).

### 3.8 Knockdown of SLC16A3 leads to diminished proliferation and invasion of lung cancer cells

**Fig. 8:**
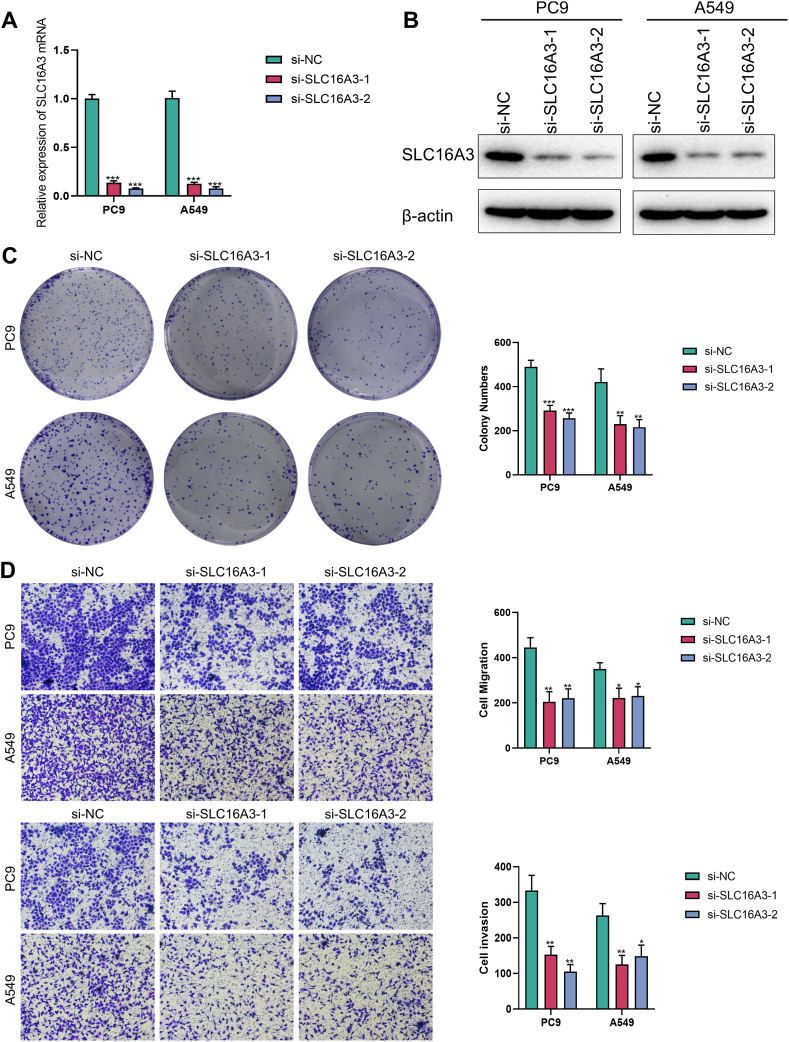
Knockdown of SLC16A3 reduced the proliferation and invasion of LUAD cell lines. (A) The knockout effect of si-SLC16A3 was confirmed at the transcriptional level using qRT-PCR. (B) The knockout effect of si-SLC16A3 was confirmed at the protein level using western blotting. (C) Representative images of colony formation in two lung cancer cell lines after knockdown of SLC16A3 and their statistical results. (D) Representative images of Transwell assays in two lung cancer cell lines after knockdown of SLC16A3 and their statistical results. Up: Migration assays; down: invasion assays.

**Fig. 9:**
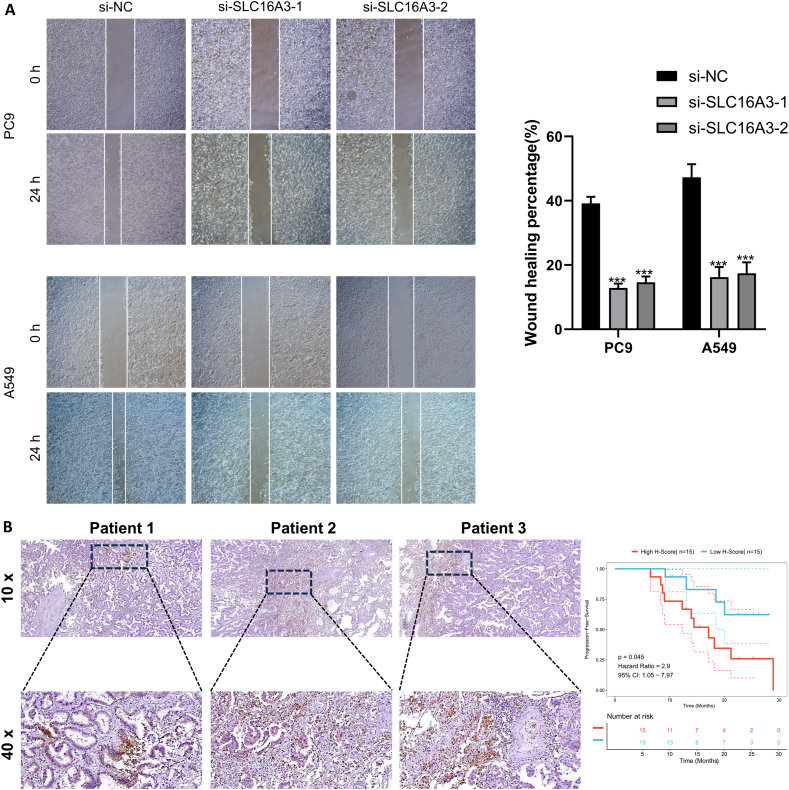
SLC16A3 is associated with poor prognosis in LUAD. (A) Representative images of wound healing assays in two lung cancer cell lines after knockdown of SLC16A3 and their statistical results. Up: PC9 cell line; down: A549 cell line. (B) Representative SLC16A3 immunohistochemical staining plots from three patients and survival curves for SLC16A3.

Considering that SLC16A3 is the most powerful risk factor (coefficient = 0.003323) in the LMS model, we subsequently downregulated the expression of SLC16A3 in LUAD cell lines to evaluate its impact on the malignant phenotype of tumors. We validated the reduction effect of these two siRNAs on SLC16A3 at the transcriptional as well as protein levels, and mRNA and protein expression of SLC16A3 were reduced in the si-SLC16A3 group (Fig. 8A and B). Firstly, we assessed the impact of SLC16A3 downregulation on the proliferation ability zof PC9 and A549 cells through colony formation experiments, and the outcomes revealed a substantial drop in the proliferative capacity of both LUAD cell lines after downregulating SLC16A3 (Fig. 8C). We evaluated the effects of SLC16A3 on PC9 and A549 cells' ability for invasion and migration via the Transwell assay. Within the chamber without matrix gel, the migratory capacity of lung cancer cell lines with downregulated SLC16A3 was significantly reduced (Fig. 8D). Furthermore, after adding matrix gel, we found that downregulating SLC16A3 significantly weakened the PC9 and A549 cells' ability for invasion (refer to Fig. 8D). Following that, we conducted a scratch wound healing experiment on serum starved PC9 and A549 cells to evaluate the impact of SLC16A3 on the migration ability of LUAD cells. The results showed that the cells with reduced SLC16A3 had significantly lower scratch wound healing capacity compared to the empty vector cells (Fig. 9A). In conclusion, the malignant phenotype of PC9 and A549 cells was weakened after downregulating SLC16A3.

### 3.9 SLC16A3 is associated with poorer prognosis in patients with LUAD

We subsequently collected surgically excised tissue sections from our LUAD patients and assessed the staining intensity of SLC16A3 by immunohistochemistry. We show SLC16A3 staining in 3 representative patients with positive SLC16A3 in LUAD (Fig. 9B). We assessed staining intensity according to H-SCORE and classified patients with LUAD whose SLC16A3 is high or low based on the median H-SCORE. When we considered the clinical data, we discovered that patients with LUAD who had high SLC16A3 expression had a considerably worse prognosis than those who had low SLC16A3 expression (Fig. 9B).

## 4 Discussion

Lung cancer has emerged as the most prevalent and deadliest malignant tumor worldwide [1]. Specifically, the incidence of LUAD has shown a progressive increase in alignment with the rise in smoking prevalence [25]. Cancer is characterized by phenotypic alterations in cells resulting from the accumulation of genetic mutations [26]. Consequently, unraveling genetic information has emerged as a pivotal aspect in comprehending the onset as well as progression pertaining to LUAD. The aberrant accumulation of lactate within the TME not only encourages tumor cells' malignant phenotype yet additionally creates an unfavorable background for immune cell growth, facilitating the evasion of immune surveillance and clearance by tumor cells [27]. Accordingly, this study centers around exploring the genetic variations within lactate metabolism pathways in LUAD. Through the identification of lactate metabolism pathway genes that profoundly affect LUAD patients' prognosis, we have developed a reliable and robust LMS model utilizing LASSO regression for risk prediction. Subsequently, we have employed diverse bioinformatics approaches to evaluate the relevance of LMS to LUAD in terms of tumor biology and immune infiltration, while also assessing its potential influence on enhancing clinical management strategies for LUAD patients. Lastly, we have experimentally confirmed in vitro that the downregulation of SLC16A3 substantially attenuates the proliferative and invasive capabilities of LUAD cell lines.

The LMS model we constructed consists of six lactate metabolism regulators. Among them, SLC16A3 exhibits the strongest risk prediction ability. SLC16A3, one of the lactate transport proteins, facilitates the transfer of lactate from cells to the extracellular space and is essential in the hypoxic cancer cells' proliferation and survival [28]. Prior research has demonstrated the prognostic potential of SLC16A3 in hepatocellular carcinoma, cholangiocarcinoma, and bladder cancer [[29], [30], [31]]. Additionally, SLC16A3 has been discovered to inhibit the inflammatory polarization of macrophages in breast cancer [32]. It has also been demonstrated that by regulating SLC16A3 in tumor cells, the TME's lactate levels can be reduced, leading to a decreased infiltration of inhibitory immune cells, and ultimately enhancing the sensitivity of tumor patients to immunotherapy [33]. Our in vitro experiments indicate the involvement of SLC16A3 in the malignant phenotype of lung adenocarcinoma cells. Our comprehensive analysis of OS in LUAD patients confirmed LMS as an independent risk element for LUAD, and its prognostic robustness was validated in an external validation cohort. Although the prediction accuracy of LMS in the GEO model is not satisfactory, the Nomogram integrating LMS and clinical features can reliably distinguish high-risk LUAD patients. In conclusion, our LMS model shows significant potential in clinical applications.

We investigated the correlation between LMS and the biological pathways and immune infiltration changes in the development of LUAD. Our findings portrayed that elevated levels of LMS were significantly linked to the activation of the cell cycle pathway. Additionally, LMS was linked to classic cancer-related pathways, for instance, P53, KRAS, and MTOR [[34], [35], [36]]. Prior investigations have revealed that the abnormal lactate accumulation supports and promotes abnormal proliferation of tumor cells. Our study showed that elevated levels of LMS indicate a high-risk group of LUAD patients who have active proliferation and a poor prognosis. Even so, there were no substantial differences in overall immune infiltration, but there was a substantial increase in M0 macrophage and M1 macrophage infiltration in high LMS patients. It is generally believed that M0 macrophages are in a resting state, while M1 macrophages represent a type of pro-inflammatory and anti-tumor macrophages [21,37]. However, our findings indicated that high LMS patients possessed a substantially worse prognosis, possibly due to abnormally active cell proliferation response and lack of effector immune cells. Interestingly, in patients with low LMS, we found a significant infiltration of memory immune cells, including plasma cells, B cells, as well as CD4 T cells. Existing studies have shown that training targeting memory immunity can enhance the long-term effectiveness of cancer immunotherapy [38]. In addition, memory T cells have the ability to receive stimuli and exhibit persistent anti-tumor immunity and immune memory, making new antigens and vaccine therapies targeting memory T cells a new direction for cancer immunotherapy [39,40].

In conclusion, we believe that patients who have low LMS might be more responsive to cutting-edge immunotherapy, and patients who have high LMS might be better candidates for conventional chemotherapy that targets the cell cycle. Our subsequent analysis confirmed this hypothesis, revealing that patients who have high LMS possess greater sensitivity to frontline chemotherapy drugs for LUAD. High LMS indicates an active cell cycle-related pathway, providing more targets for traditional chemotherapy drugs and improving treatment sensitivity [41]. Conversely, we found that patients with low LMS have more immune-related pathways, indicating immune cell enrichment and potential sensitivity to most immunotherapies. Additionally, our evaluation using the TIDE algorithm confirmed the potential responsiveness of patients having low LMS to immune checkpoint inhibitors. Finally, in the lung cancer immunotherapy cohort, we confirmed that patients who have low LMS have significantly better survival. In larger-scale melanoma and bladder immunotherapy cohorts, LMS can still be used for risk stratification, and immunotherapy may work better for patients with lower LMS. As a result, our LMS model can offer useful direction for creating clinical intervention plans.

We acknowledge that there are still some limitations in the existing analysis. Firstly, we are unable to observe the dynamic changes in LMS before and after chemotherapy intervention in the sequencing samples from the online database. Secondly, the prediction of chemotherapy drug sensitivity is computational, and it requires controlling the clinical cohort of chemotherapy regimens to verify the actual predictive efficacy of LMS in chemotherapy. Lastly, our in vitro experiments only confirmed the impact of SLC16A3 on the malignant phenotype of LUAD cell lines. Future research needs to further explore its mechanism of action through in vivo experiments.

## 5 Conclusion

Our research is based on genetic alterations in the lactate metabolism pathway and has developed an LMS model applicable for risk stratification of LUAD patients. LMS can identify high-risk patients with active cell cycle and provide guidance for chemotherapy and immunotherapy in LUAD.

## Availability of data and material

The article and supplementary materials contain the original contributions made during the study; for additional information, contact the corresponding author.

## Ethics statement

The Human Ethics Committee of Zhongda Hospital, Southeast University, approved the experimental protocol, which was developed in accordance with the Helsinki Declaration's ethical guidelines (approval 2022ZDSYLL303-P01). Participants, either as individuals or as guardians, gave written informed consent.

## Funding

This work was supported by the Jiangsu Health of the Elderly Foundation (No.LKM2022085).

## CRediT authorship contribution statement

**Jing Zhang:** Writing – original draft. **Yun Bao:** Resources. **Yang Li:** Investigation. **Xin Shi:** Validation. **Xiangyu Su:** Resources. **Xuejun He:** Writing – review & editing.

## Declaration of competing interest

The authors declare that they have no known competing financial interests or personal relationships that could have appeared to influence the work reported in this paper.
